# UXT, a novel MDMX-binding protein, promotes glycolysis by mitigating p53-mediated restriction of NF-κB activity

**DOI:** 10.18632/oncotarget.3770

**Published:** 2015-05-07

**Authors:** Min Qi, Suthakar Ganapathy, Weiqi Zeng, Jianglin Zhang, John B. Little, Zhi-Min Yuan

**Affiliations:** ^1^ Department of plastic surgery, Xiangya Hospital, Central South University, Changsha, Hunan, PR China; ^2^ Department of Genetics and Complex Diseases, Harvard T.H Chan School of Public Health, Boston, MA, USA; ^3^ Department of Dermatology, Xiangya Hospital, Central South University, Changsha, Hunan, PR China

**Keywords:** p53, NF-κB, MDMX, UXT

## Abstract

The importance of stress-induced p53 activation has been extensively investigated and well established. How the basal activity of p53 prevents carcinogenesis, however, remains incompletely understood. We report the identification of a novel p53 inhibitor, UXT, which binds to MDMX and suppresses the basal activity of p53. Interestingly, human TCGA database indicates that the *UXT* gene is frequently amplified in human sarcoma where p53 mutation is rare. We thus used sarcoma as a model to show that UXT acts as an oncogene promoting cell proliferation *in vitro* and tumor progression *in vivo*. A screening of 10 major cellular pathways uncovered that UXT-mediated p53 inhibition results in an activation of NF-κB, leading to induction of glycolysis. While elevated glycolytic metabolism provides growth advantage it also renders UXT expressing sarcoma cells heightened sensitivity to glycolysis inhibition. Altogether, our data demonstrate a crucial role for the basal activity of p53 in restriction of NF-κB. By impeding such an activity of p53, UXT unleashes the oncogenic activity of NF-κB resulting in induction of glycolysis fueling carcinogenesis.

## INTRODUCTION

In response to diverse stress signals, p53 is readily activated inducing a host of cellular effects including senescence and cell death, amongst others [1]. Although the p53-mediated stress responses are important to prevent tumor development, its activity has to be tightly regulated to avoid unnecessary pathological consequences [1]. Elaborate mechanisms exist to maintain an appropriate homeostasis of p53 activity. While many players are reported to play a role, abundant evidence indicates that MDM2 and MDMX are the principal regulators keeping p53 at a low basal level [2]. MDM2 and MDMX function primarily together as a complex to inhibit p53 by directly blocking the transactivation domain of p53 and by targeting p53 for ubiquitination/degradation [3, 4]. Many stress signals impinge on the MDM2/MDMX complex and disable their interaction with p53 resulting in p53 activation [5]. Whereas insufficient MDM2/MDMX activity can cause p53-dependent cytotoxicity, excess activity of this inhibitory complex can be oncogenic. Indeed, both MDM2 and MDMX are well-established oncogenes because they are frequently overexpressed in many human cancers where the *p53* gene is rarely mutated. Although MDM2 and MDMX are potent p53 inhibitors, DNA damage-induced p53 activation in MDM2 and MDMX overexpressing cells remains most intact [6], implicating a critical importance of downregulating the basal p53 activity to the oncogenic function of MDM2 and MDMX. Interestingly, as one of the most studied proteins, the knowledge regarding to the importance of the basal steady state level of p53 to its role as a tumor suppressor remains limited.

The transcription factor NF-κB regulates various genes important for the immune response, cell proliferation, and cell survival in response to various cellular stresses such as cytokine activation, oxidative stress, and infectious diseases [7, 8]. During the immune response, cells consume large amounts of glucose and primarily use aerobic glycolysis to rapidly produce enough energy to meet the bioenergetic demands of cellular proliferation and survival [9]. The NF-κB pathway has been shown to stimulate aerobic glycolysis by upregulating the expression of GLUT-3 and HIF1α [10, 11], mediating the metabolic response critical for cell function and survival.

The NF-κB pathway is often deregulated in human cancer leading to an excess activity that is largely oncogenic [7]. Dynamic crosstalk between the p53 and NF-κB pathways has been widely observed. Although this crosstalk is highly context dependent and has been shown to function either as antagonistic or cooperative between the two pathways, p53 and NF-κB are considered to overall function against one another; pro-death versus pro-survival [12, 13]. In the context of cellular metabolism, p53 favors oxidative phosphorylation whereas NF-κB stimulates glycolysis.

In this report, we describe the identification of UXT as a novel MDMX-interacting protein. UXT binds to and stabilizes MDMX resulting in reduction of the basal steady state p53 activity. Of interest is the finding that NF-κB activity was selectively upregulated upon p53 inhibition by UXT. Using a combination of metabolomic and genetic approaches, we demonstrated that NF-κB activation induced glycolytic metabolism fueling cancer cell growth and survival. In support of TCGA data showing that the *UXT* gene is frequently amplified in human cancers, our study uncovers a novel mechanism of oncogenic role of UXT in suppression of basal p53 activity causing NF-κB-mediated induction of glycolysis and carcinogenesis.

## RESULTS

### Identification of UXT as a novel MDMX binding protein

**Figure 1:**
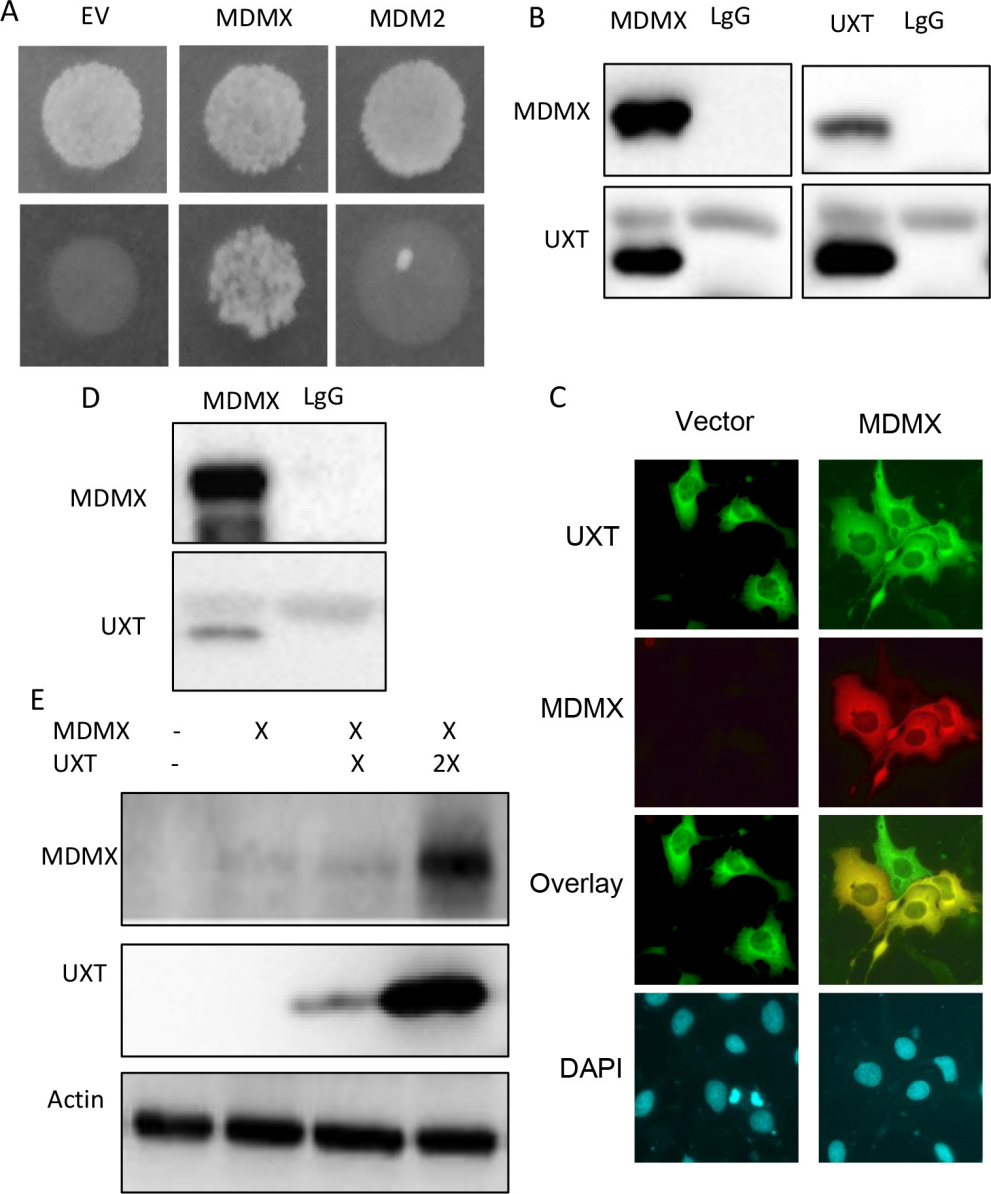
UXT binds to and stabilizes MDMX **A.** A result of a yeast 2-hybrid assay indicating a positive clone pull out by a bait of MDMX but not MDM2. **B.** 293 cells were coexpressed with plasmids encoding a Flag-UXT and MDMX. The cells were harvest 24 h after transfection and subjected to reciprocal immunoprecipitations with an anti-Flag (M2 Sigma) or MDMX (Bethyl lab), with lgG included as a control. The immunoprecipitates were analyzed by Western analysis using anti-Flag or anti-MDMX antibodies. **C.** U2OS cells were transfected with either GFP-UXT or RFP-MDM alone to together. The cells were fixed 24 h later. Images were taken under a fluorescent microscope and representative images including overlay ones are shown. **D.** U2OS cells were subjected to anti-MDMX immunoprecipitation with LgG included as a control. The immunoprecipitates were immunoblotted with either anti-MDMX or anti-UXT. **E.** 293 cells were transfected with a control vector or 1 μg MDMX with 1 or 3 μg of Flag-UXT. The cells were harvested 24 h later and analyzed by Western blot with the indicated antibodies.

As the principal negative regulators of p53, MDMX and MDM2 form a MDM heterocomplex that works together in p53 control. The MDM complex inhibits p53 either as an E3 ligase targeting p53 for ubiquitination/degradation or directly masking the transactivation domain of p53. Given the importance of the complex in p53 control, any protein that interacts with either MDM2 or MDMX may affect their ability to inhibit p53. We tested this hypothesis by conducting a yeast 2-hybrid screening to search for MDMX-binding partners. We chose MDMX over MDM2 because the later associates with DNA, which led to numerous false positives (not shown). The screening identified an understudied protein, UXT (Figure 1A). Of interest is that mining of TCGA database revealed UXT as a gene frequently overexpressed in human sarcoma (Supplementary Figure 1) where p53 inactivation is usually caused by a heightened activity of its inhibitors because the p53 gene mutation is rare [1]. We hypothesized that UXT might contribute to negative regulation of p53 via its binding to MDMX. We tested this hypothesis by first confirming the interaction between UXT and MDMX. 293 cells co-expressing UXT with MDMX or MDM2 were subjected to a reciprocal IP-Western analysis. The result indicated a clear binding between UXT and MDMX (Figure 1B). The IP-Western data were further corroborated by immunostaining, which revealed an overt colocalization of the 2 proteins (Figure 1C), indicative of an association between UXT and MDMX. The association between UXT and MDMX was also observed with endogenously expressed protein (Figure 1D). Protein-protein interaction often affects the protein stability of each binding partner. We tested this possibility by coexpression of MDMX with an increasing amount of UXT, which indeed resulted in a dose-dependent increase in MDMX protein abundance (Figure 1E). The data altogether indicated that UXT binds to and stabilizes MDMX.

### UXT negatively regulates p53 activity enhancing cell proliferation

**Figure 2:**
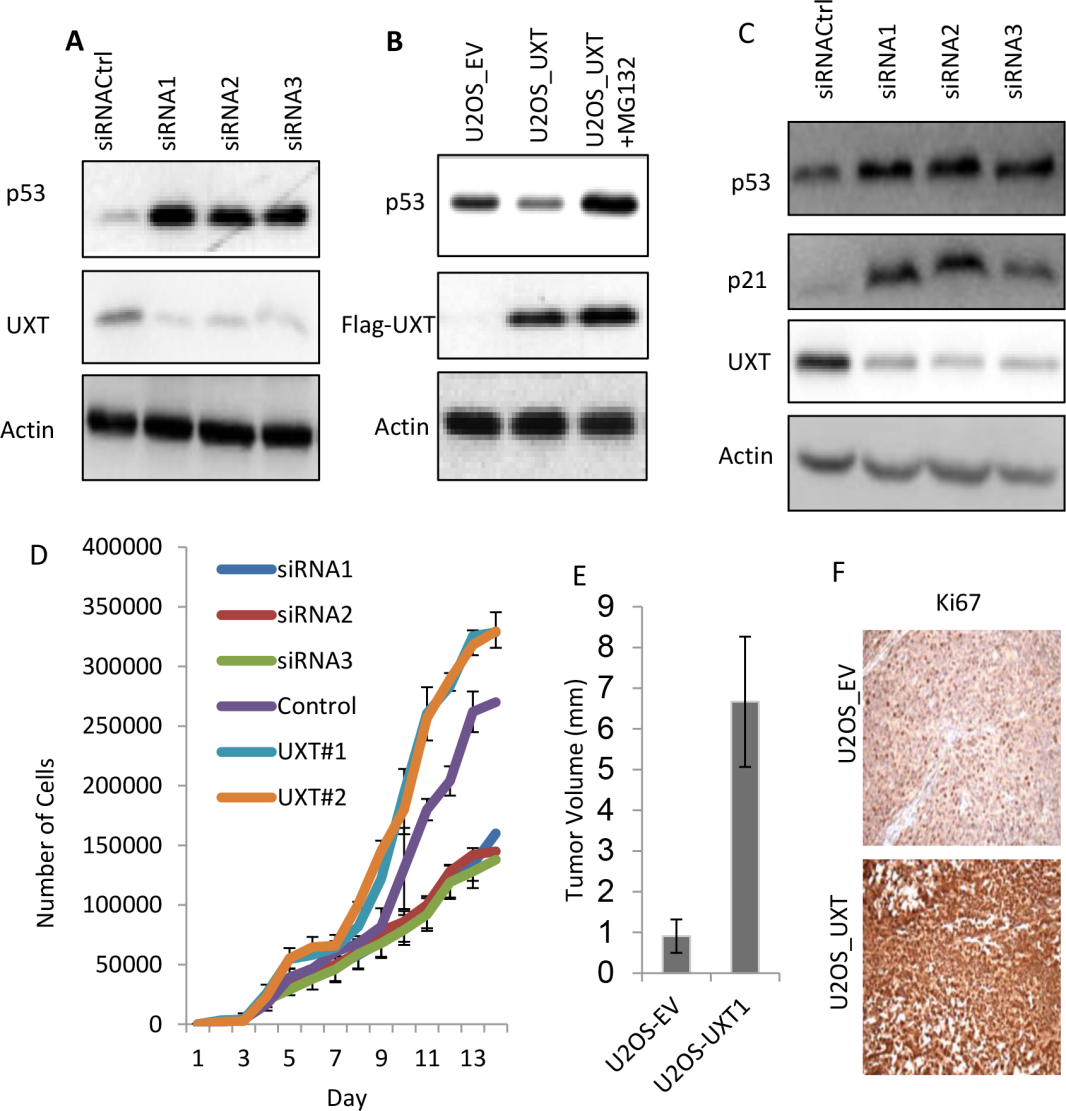
UXT functions as an oncogene via suppression of p53 activity **A.** U2OS cells were transfected with either control siRNA (siRNACtrl) or 2 independent siRNA sequences targeting different region of the *UXT* gene. The cells were harvested 48 h later and analyzed for protein expression by Immunoblot using the indicated antibodies. **B.** UOS cells stably expressing an empty vector (EV) or UXT were established. The U2OS_UXT cells were treated with or without MG132 (10 μM) for 6 h prior to harvesting for Western analysis using the indicated antibodies. **C.** U2OS cells were transfected as in A and harvest 50 h later for Western analysis using the indicated antibodies. **D.** U2OS cells transfected with each of siRNA(1, 2 or 3) or 2 different patches of UXT overexpressing U2OS cells (UXT#1 or #2) were measured the rate of cell proliferation using a proliferation assay kit (Gibco). **E.** Balb C nude mice (4–6 weeks) were subcutaneously implanted with 2 × 10^6^ U2OS_EV or U2OS_UXT cells. The tumors were harvested 8 weeks later and the volumes were measured. The numbers are averages from a group of 5 mice ± standard deviation. **F.** The harvested tumor tissues were subjected to immunohistochemistry staining with an anti-Ki67 antibody. The representative images were shown.

Given that MDMX is a negative regulator of p53, UXT-mediated stabilization of MDMX would predict this protein as an inhibitor of p53. We employed methods of over- and under-expression of UXT to test this possibility. siRNA-mediated knockdown of UXT was associated with a considerable increase in p53 abundance (Figure 2A). The use of multiple siRNA sequences of UXT indicated that p53 activation was specifically caused by UXT knockdown. In contrast to the effect of UXT depletion, UXT overexpression was associated with a decrease in p53 level, which seemed to be a result of increased turnover because inhibition of protein degradation by MG132, a proteasome inhibitor, recovered the p53 level (Figure 2B). To examine whether the increased p53 protein abundance correlated with its transcriptional activity. We determined the expression of p21, a target gene of p53 and found a marked induction of p21 expression upon UXT knockdown (Figure 2C). This increase of p21 in UXT-depleted cells was p53-dependent as siRNA-mediated p53 knockdown or E6-mediated p53 degradation completely abolished this effect of UXT (Supplementary Figure 2).

**Figure 3:**
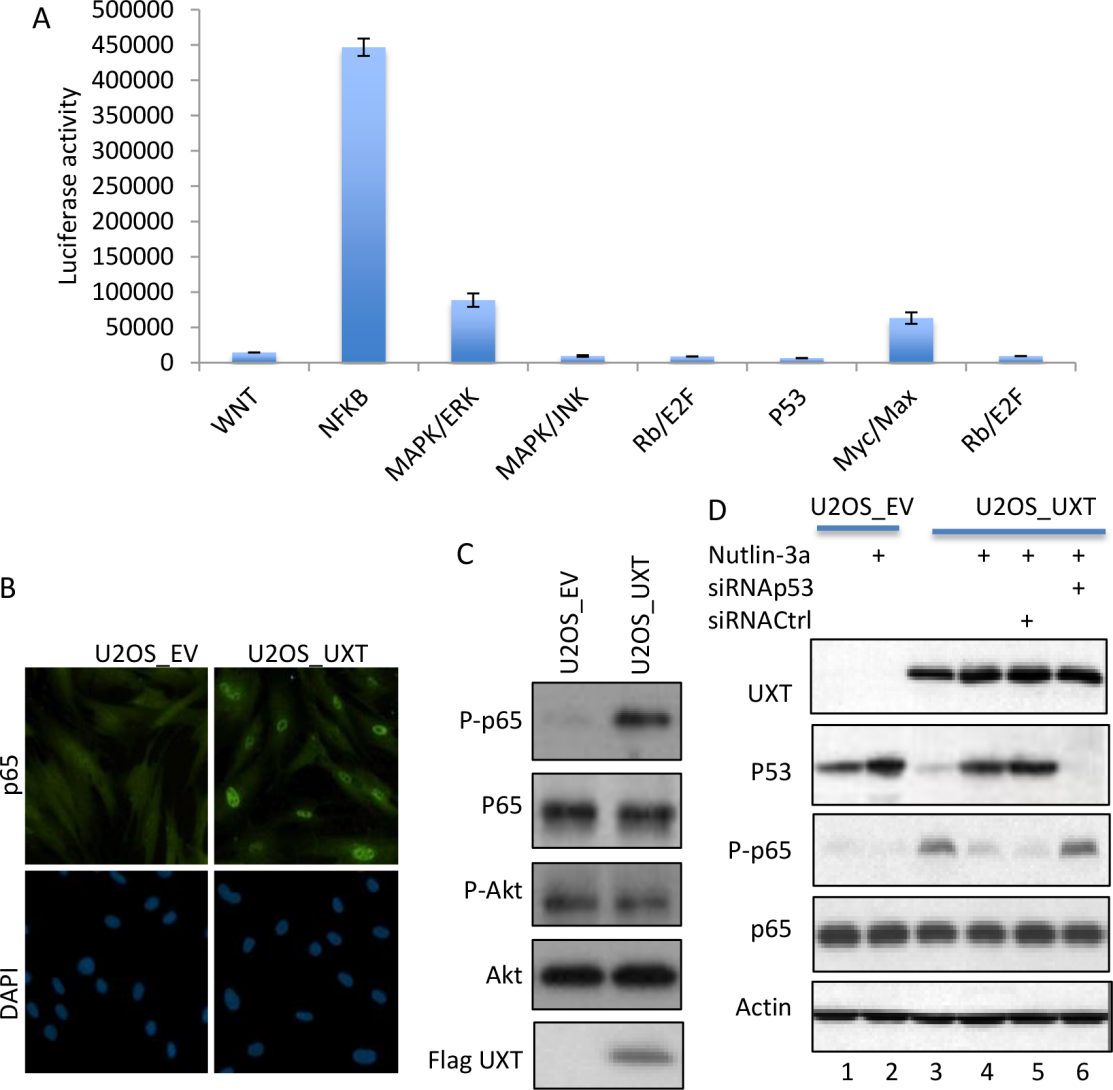
UXT-induced p53 inhibition caused activation of NF-B **A.** U2OS_UXT cells were subjected to Cignal Finder™ 10-Pathway Reporter Arrays (Qiagen). The results are from 3 independent experiments performed in triplicates. The numbers are means ± SD. **B.** U2OS_EV or U2OS_UXT cells were analyzed by immunostaining with an anti-p65 (Cell Signaling, MA) co-stained with DAPI. Representative images are shown. **C.** Cell lysates prepared from U2OS_EV or U2OS_UXT cells were analyzed by Immunoblot with the indicated antibodies. **D.** U2OS_EV or U2OS_UXT cells that were transfected with either siRNACtrl or siRNAp53 were treated with or without Nutlin-3a (10 μM 3 h). The cells were harvested and subjected Western analysis using the indicated antibodies.

We went on to investigate the biological consequence to UXT-mediated p53 regulation by monitoring its effect on cell proliferation, considering the well recognized growth inhibitory activity of p53. In line with the induction of p21 in response to the expression of UXT siRNA, reduced expression of UXT was associated with a marked decrease of the rate of cell proliferation. Conversely, stable overexpression of UXT led to an increase in cell proliferation. Two separate batches of UXT expressing cell lines exhibited a higher rate of proliferation than control cells (Figure 2D). We went on to carry out mouse xenograft experiments to test the *in vivo* oncogenic activity of UXT. Consistent with the data of cell proliferation, U2OS_UXT cells developed into significantly larger tumors than U2OS_EV cells (Supplementary. Figure 3 & Figure 2E). Immunohistochemistry analysis of Ki67, a commonly used proliferation marker, indicated a higher rate of cell proliferation in U2OS_UXT cells than U2OS_EV cells (Figure 2F). Taken together, our data revealed that UXT inhibits p53 activity via binding to MDMX promoting cancer cell proliferation *in vitro* and tumor development *in vivo*. Such an oncogenic role of UXT is consistent with its overexpression in human cancer.

### UXT-mediated p53 suppression was associated with NF-κB activation

Having shown UXT-induced growth stimulation, we explored the underlying mechanism by performing a screening of the 10 cellular signaling pathways via a luciferase-based assay. Interestingly, UXT overexpression was associated with a selective increase in the activity of the NF-κB pathway, whereas the AKT, MAP and other cell growth promoting pathways were not significantly altered. We further verified the effect of UXT on the NF-κB activity by examining p65 subcellular distribution and p65 phosphorylation, 2 commonly used markers of NF-κB activity [7]. Indeed, a clear increase in nuclear localization of p65 was evident when U2OS_UXT expressing cells were compared with the U2OS_EV cells (Figure 3B). In support of increased NF-κB activity, Immunoblot with a phosphor-specific antibody revealed that p65 was markedly phosphorylated in U2OS_UXT but not U2OS_EV cells (Figure 3C). Little change of Akt phosphorylation was detected, consistent with the result obtained from pathway screening indicating a selective stimulation of NF-κB activity in U2OS_UXT cells. We went on to explore the mechanism by which NF-κB is activated by UXT expression. Available information indicates an antagonistic interaction between p53 and NF-κB [11]. Given that merely loss of p53 function can lead NF-κB activation [12], we asked whether the compromised function of p53 in U2OS_UXT cells could be responsible for the elevated NF-κB activity. We tested this question using a small molecular p53 activator Nutlin-3a, which disassociates the binding of MDM2 to p53 [5]. Treatment of U2OS_UXT cells with Nutlin-3a resulted in robust p53 activation (Figure 3D, lane 2), as expected. Remarkably, the increased p53 activity was associated with diminished NF-κB activity (Figure 3D, lane 4). This effect appeared to be p53-dependent because the NF-κB activity was not reduced by nulin-3a when the expression of p53 was depleted by siRNA (Figure 3D, lane 6). Together, the results indicated an elevated NF-κB activity in U2OS_UXT cells because of UXT-mediated p53 inhibition, consistent with an antagonistic interaction between p53 and NF-κB [12, 13].

### NF-κB promotes glycolytic metabolism

**Figure 4:**
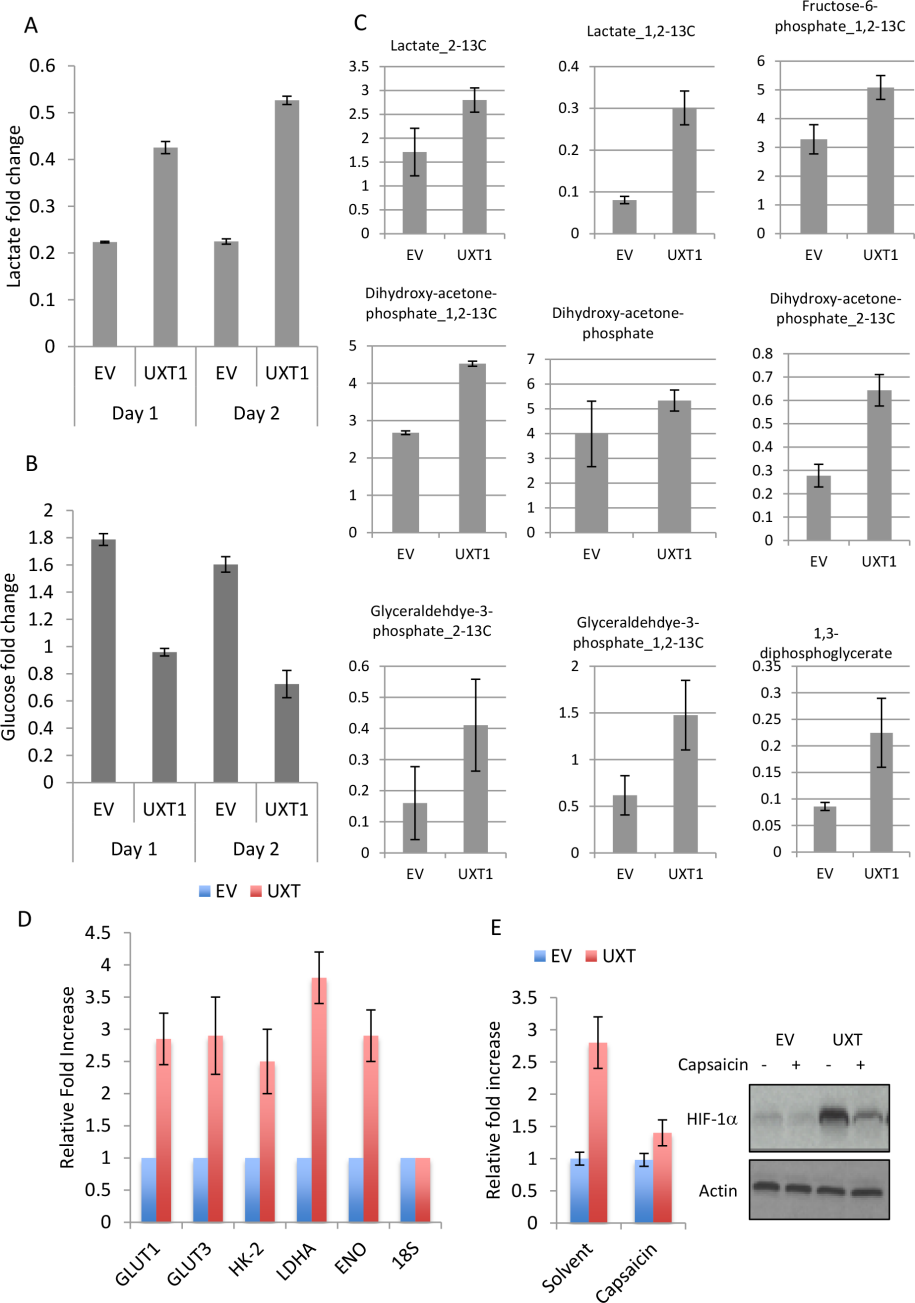
UXT induces glycolytic metabolism by NF-κB-dependent upregulation of HIF-1α Cell culture media were collected from an equal number cells. The concentrations of lactate **A.** and glucose **B.** were determined by a colorimetric kit (BioVision). The numbers are means ± SD from 3 experiments performed in triplicates. **C.** U2OS_EV or U2OS_UXT cells were incubated with [1, 2-^13^C]-glucose for 15 min prior to metabolite extraction and targeted LC-MS/MS analysis. The ratio of ^13^C labeled to unlabeled (^12^C) metabolites was measured by LC-MS/MS are presented as mean ± SD over 3 independent samples. Metabolites with *P* values < 0.05 for pair-wise comparisons are shown. **D.** U2OS_EV or U2OS_UXT cells were harvested and mRNA isolated. Transcripts of the indicated genes were determined by quantitative RT-PCR. Data are means ± SD from 3 experiments performed in triplicates. **E.** U2OS_EV or U2OS_UXT cells were treated with or without Capsaicin (100 μM 3 h). qRT-PCR or Western determined the expression levels of HIF-1α transcripts and protein, respectively.

The transcription factor NF-κB plays an important role in multiple cellular processes, including immune signaling, inflammation, proliferation and survival [7]. In cancer cells, NF-κB activation is frequently associated with increased proliferation and survival. Interestingly, we observed that the media color of U2OS_UXT culture turned yellowish much more rapidly than that of U2OS_EV cells (Supplementary. Figure 4), suggesting increased production of acidic metabolites. In line with this observation was the finding that NF-κB plays a key role in regulation of cellular metabolism. Increased NF-κB activity stimulates glycolysis by inducing the expression of glucose transporter and glycolytic genes, directly or indirectly [9, 10]. We asked whether the yellowish media color was caused by increased rate of glycolysis. Measurement of the extracellular concentration of glucose and lactate (the major product of glycolysis) revealed a significant decrease in glucose whereas an increase in the lactate level in UXT expressing cells relative to the control cells (Figure 4A – 4B), consistent with an increased rate of glycolysis. To substantiate this finding we performed glucose flux analysis and indeed detected a significant increase in the rate of glycolytic metabolism, as evidenced by overt accumulation of a number of glycolytic metabolites (Figure 4C). To corroborate the metabolomics data, we measured the level of transcripts of metabolic genes. Interestingly, the expression of multiple glycolytic genes including *GLUT-1* & *3*, *HK-2* & *3, LDHA* and *ENO* was significantly increased in UXT expressing cells when compared with the control cells (Figure 4D). The induction of so many glycolytic genes was unexpected since *GLUT-3* is the only known target gene of NF-κB [7). Previous studies showed that NF-κB could transcriptionally induce the expression of HIF-1α, a master transcription factor for multiple glycolytic genes [9, 10]. We thus measured the expression of HIF-1α and detected a considerable induction of this transcription factor at both the transcript and protein levels in UXT expressing cells (Figure 4E). In line with the NF-κB -mediated effect, the increase in HIF-1α was almost completed abrogated when a specific inhibitor of NF-κB (Capaisacin) was added to the cell culture. The result together showed that the increased activity of NF-κB in UXT overexpressing cancer cells induced glycolysis via upregulation of HIF-1α.

### NF-κB-induced glycolysis contributes to UXT-enhanced cell proliferation and survival

**Figure 5:**
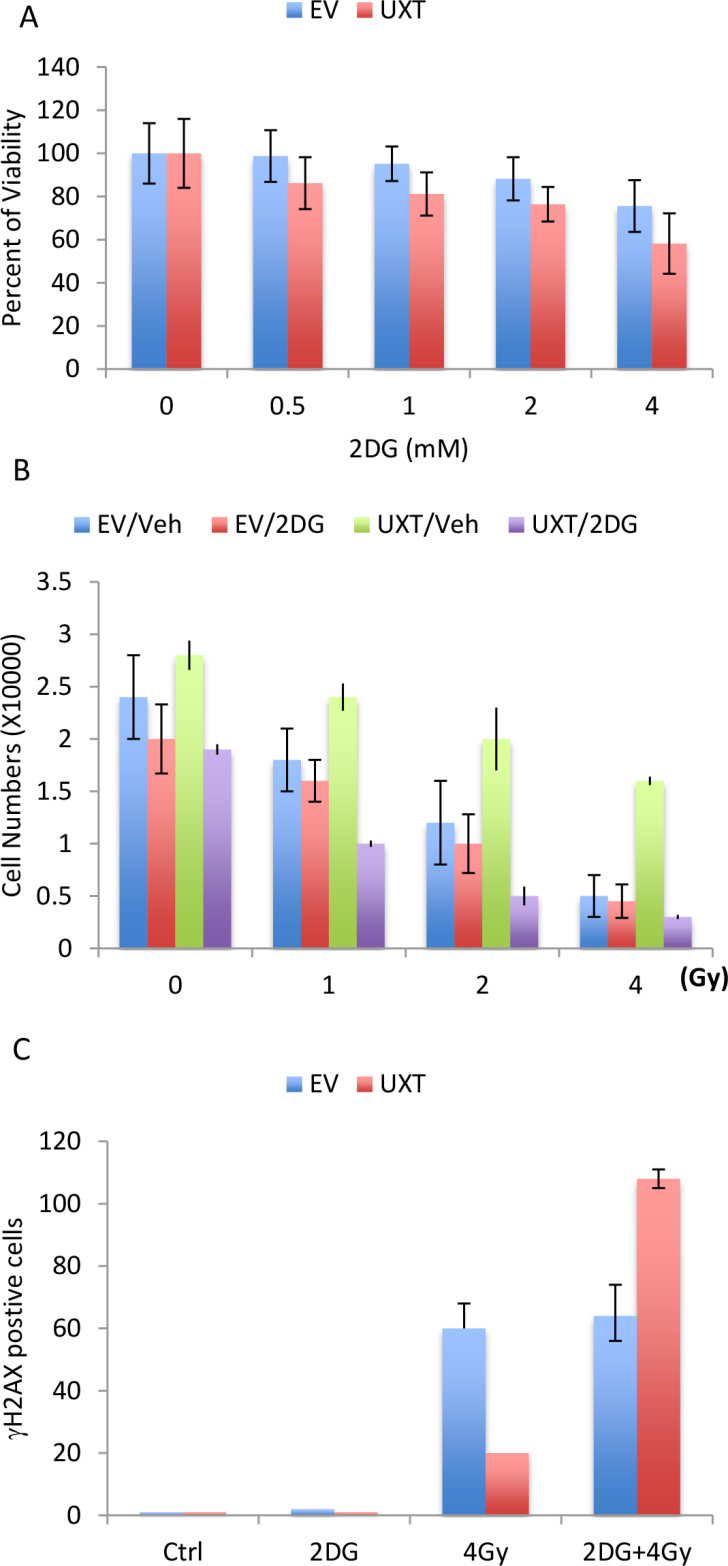
U2OS_UXT cells depend on glycolysis for cell survival **A.** U2OS_EV or U2OS_UXT cells were treated with or without 2-DG at the indicated concentration for 48 h. Cell viability was determined and the data are means ± SD from 3 experiments performed in triplicates. **B.** U2OS_EV or U2OS_UXT cells were pre-treated with vehicle (Veh) or 2-DG (2mM) for 6 h followed by irradiation at the indicated dose. Cell numbers were determined 72 h after irradiation. Data are means ± SD from 3 experiments performed in triplicates. **C.** U2OS_EV or U2OS_UXT cells were pre-treated with vehicle (Veh) or 2-DG (2mM) for 6 h followed by irradiation at a dose of 4Gy. The cells were fixed 1 h after irradiation and stained with γH2AX. The numbers of γH2AX-positive cells were counted from 5 random fields. Data are means ± SD from 3 experiments performed in triplicates.

Given the importance of elevated glycolysis, or “Warburg effect” in cancer cell proliferation and survival, the UXT-mediated induction of glycolysis prompted us to examine its role in cell proliferation. We tested whether UXT-dependent cell proliferation could be affected by inhibition of glycolysis via the use of a specific inhibitor of glycolysis, 2-deoxyglucose. The result indeed revealed an important role of glycolysis in UXT-induced cell proliferation. Addition of 2DG was associated a dose-dependent suppression of cell proliferation and importantly, the inhibitory activity of 2DG was much stronger in UXT overexpressing cells than in the control cells (Figure 5A), consistent with a notion that UXT stimulates cell proliferation via at least in part promoting glycolytic metabolism. We next investigated the contribution of glycolysis to cancer cell sensitivity to cancer therapy. When compared with U2OS_EV cells, U2OS_UXT cells exhibited increased resistance to irradiation-induced killing (Figure 5B). Interestingly, inhibition of glycolysis with 2DG considerably sensitized U2OS_UXT cells to irradiation-induced cell death whereas U2OS_EV cells were much less affected (Figure 5B). To substantiate the observation, we measured irradiation-induced γH2AX, a surrogate marker of DNA damage. In line with the cell survival results, U2OS_UXT cells were more resistant to irradiation as evident by significantly fewer numbers of γH2AX-positive cells than U2OS_EV cells induced by 4Gy-irradiation (Supplementary Figure 5). However, treatment with 2DG was associated with a much greater increase in γH2AX-positive cells in U2OS_UXT cells than U2OS_EV cells (Figure 5C), indicating that inhibition of glycolysis preferentially sensitized U2OS_UXT cells over U2OS_EV cells. The results altogether support an important role of glycolysis in supporting survival of UXT expressing cells.

## DISCUSSION

In this study, we identified UXT as a novel MDMX interacting protein and the binding of UXT resulted in stabilization of the MDMX protein and consequent inhibition of p53 activity, uncovering a previously unappreciated oncogenic activity of UXT. The biological significance of UXT-mediated p53 suppression is underscored by the finding that the *UXT* gene is frequently amplified in several types of human cancer according to the TCGA database. Among them, human sarcoma is of particular interest because the *p53* gene in this type of cancer is usually not mutated [1], implicating UXT-dependent p53 inhibition as a novel mechanism of p53 inactivation in human sarcoma. Using a combination of loss-of-function and gain-of-function approach, we provided multiple lines of *in vitro* and *in vivo* evidence demonstrating an oncogenic role of UXT in sarcoma progression.

Recent studies showed that a diminished p53 function caused a constitutive activation of NF-κB [12]. The basal p53 level in UXT expressing cells was considerably down regulated because of increased degradation (Figure 2A), likely caused by the elevated level of MDMX, which forms a heterocomplex with MDM2 targeting p53 for ubiquitination/degradation [3, 4]. In line with the antagonistic relationship between p53 and NF-κB, the diminished p53 activity in UXT-expressing cancer cells was associated with an elevated NF-κB activity, which was initially uncovered by the pathway screening and subsequently validated by examining p65 nuclear distribution and phosphorylation. We provided evidence demonstrating that UXT-mediated p53 inhibition was indeed responsible for the increased activity of NF-κB. It has been reported that p53 could also antagonize m-TOR activity [14]. We, however, detected little change of AKT activity in UXT cells, indicative of a selective activation of NF-κB. Together with the published work [12], our data underscore a crucial role of the basal p53 activity in curbing oncogenic NF-κB activity. P53 inactivation would not only result in impediment of its canonical tumor suppressive activities such as cell cycle arrest, senescence or apoptosis, but also unleashing its restraint on NF-κB leading to the activation this oncogenic transcription factor.

NF-κB promotes cancer cell proliferation and survival by controlling the expression of a large number of genes, which affect diverse cellular processes [7]. We provide compelling evidence linking NF-κB activity to tumor metabolism, or increased aerobic glycolysis observed in UXT-overexpressing cancer cells. Apart from directly upregulating *GLUT-3* expression, we showed that NF-κB also induced transcriptionally HIF-1α, a master transcription factor that regulates almost every genes of the glycolytic pathway. Indeed, UXT-expressing cells exhibited increased expression of multiple glycolytic genes, offering genetic evidence for the elevated glycolysis.

Consistent with the importance of increased glycolytic metabolism or the Warburg effect in cancer cell proliferation and survival, we demonstrated that UXT expressing cells depended on glycolysis for proliferation and survival, displaying heightened susceptibility to glycolytic inhibition. The increased sensitivity by inhibition of glycolysis could be due to reduced DNA damage repair since we recently showed that inhibition of glycolysis in cancer cells resulted in chromatin compaction, impeding the assess of DNA damage repair proteins [13]. Given the challenge of targeting NF-κB due to its diverse effects, the elevated glycolysis may serve as a better therapeutic target.

In summary, we showed that UXT binds to and stabilizes MDMX resulting in p53 inhibition. Using sarcoma as a model, we demonstrated that UXT-mediated inhibition of the basal p53 activity resulted in NF-κB activation, which promoted cell proliferation and survival by stimulating glycolysis. Our study offers molecular mechanistic data supporting an oncogenic role of UXT revealed by human TCGA database. The increased sensitivity of UXT expressing cancer cells to inhibition of glycolysis may carry important therapeutic implication.

## MATERIALS AND METHODS

### Reagents

All chemicals were purchased from Sigma-Aldrich (St Louis, MO).

### Cell culture

Human osteosarcoma U2OS cells and 293 cells were purchased from American Type Culture Collection (ATCC, Manassas, VA). Cells were maintained in DMEM (Corning cellgro). All the media were supplemented with 10% fetal bovine serum and 1% penicillin-streptomycin-gentomycin (Invitrogen) in a humidified atmosphere at 37°C and 5% O2, 5% CO2.

### Immunofluorescence

For immunofluorescence, U2OS cells were fixed, permeabilized and blocked followed by the incubation with primary antibodies overnight. The slides were then incubated with, DAPI, rabbit Alexa Flour 488, mouse Alexa Flour 594. Nikon TE2000 microscope and NIS elements software were used for imaging.

### SiRNA transfection

SiRNAs were purchased from Sigma-Aldrich (St Louis, MO). Multiple sequences of SiRNAs were used for p53 and UXT. SiRNAs were reversely transfected using Lipofectamin RNAiMAX (Invitrogen) as per the manufacturer's instruction. SiGL2 was used as the negative control.

### RNA isolation and quantitative RT-PCR

Total RNA was isolated using Trizol Reagent (Invitrogen) according to the manufacturer's instruction. 1 μg of total RNA was used to make cDNA (iScript cDNA synthesis kit, Bio-Rad), following the manufacturer's instruction, which was subsequently used for the amplification by quantitative RT-PCR using Applied Biosystem StepOnePlus in the presence of SYBR Green JumpStart (Sigma-Aldrich, St Louis, MO). Ribosomal RNA 18S was used as endogenous normalization control. The n-fold change in mRNAs expression was determined on the basis of ΔΔ*C*t value. All assays were performed in triplicate.

### Immunoblotting

Cells were washed with ice cold phosphate buffered saline and then lysed using lysis buffer (20 mM Tris-HCl, pH 7.5, 5mM EDTA, 150 mM NaCl, 1% Nonidet P-40, I mM Na3VO4, 1mM PMSF, 0.1% protease inhibitor) for 45 minutes on ice. The cell extracts were centrifuged at 12, 000 RPM for 15 minutes at 4°C. Equal amounts of cell lysates were separated by SDS-PAGE. The separated proteins were transferred to nitrocellulose membrane and immunoblotted using indicated antibodies. Antibodies were from BD Biosciences (HIF-1α), Cell Signaling Technology (P-p65, P-Akt), abcam (p53, p21, p65, Akt), Sigma-Aldrich (Flag, β-actin). The protein bands were developed using HRP-conjugated secondary antibodies with ECL-chemiluminescent reagent.

### Metabolic flux analysis

Flux studies were performed according to a published protocol (18). U2OS-EV or U2OS-UXT cells were washed thoroughly with glucose free medium and incubated the cells with medium containing 10mM 1:1 mixture of D-[1, 2-^13^C]-glucose and unlabeled D-glucose for 15 min. Metabolites were extracted on dry ice with 80% methanol. The metabolites were dried under nitrogen and re-suspended in 20 μL of water for liquid chromatography-mass spectrometry (LC-MS) analysis.

### Cell viability and FACs analysis

Cell viability was assessed using the trypan-blue exclusion assay. The percentages of viable cells were counted as follows:

$$\text{Viable cells (\%)}=\frac{\text{Total number of viable cells per ml of aliqout}}{\text{Total number of cells per ml of aliquot}}\times 100$$

An annexin V apoptosis kit (Biovision, #K101–100) was used for the FACS-based assay as per manufacturer's instructions

### Animal experiment

All the procedures on animals were conducted in accordance with the guidelines for the Institutional Animal Care and Use Committee (IACUC) at Harvard T.H. Chan School of Public Health. Mice used in this study were housed under pathogen-free conditions and maintained in 12-h light/12-h dark cycle, with food and water supplied *ad libitum*. U2OS cells (3×10^6^ cells mixed with Matrigel, Bedton Dickinson, Bedford, MA) cells in a final volume of 100 μL were injected into the flank region of 4–6 weeks old Balb/c*^nu/nu^* (Harlan laboratories) mice. When tumors reached 0.1 cm, mice were randomized into different groups for treatments.

### Histological analysis

Tissues were fixed with 10% formalin, embedded in paraffin and sectioned. Hematoxylin and eosin staining were performed according to standard procedure.

### Statistical analysis

In-vitro experiments were repeated at least three times. Two-way ANOVA was used for the statistical calculation. Mann-Whitney *U*-test was used for comparisons between different groups.

## SUPPLEMENTARY FIGURE LEGENDS


