# Supplementary material for: UXT, a novel MDMX-binding protein, promotes glycolysis by mitigating p53-mediated restriction of NF-κB activity
Source: Oncotarget. 2015 May 7;6(19):17584–93. doi: 10.18632/oncotarget.3770 (PMC4627330; doi:10.18632/oncotarget.3770)
Supplement: Supplementary file 1 [file oncotarget-06-17584-s001.pdf]

SUPPLEMENTARY FIGURE LEGENDS

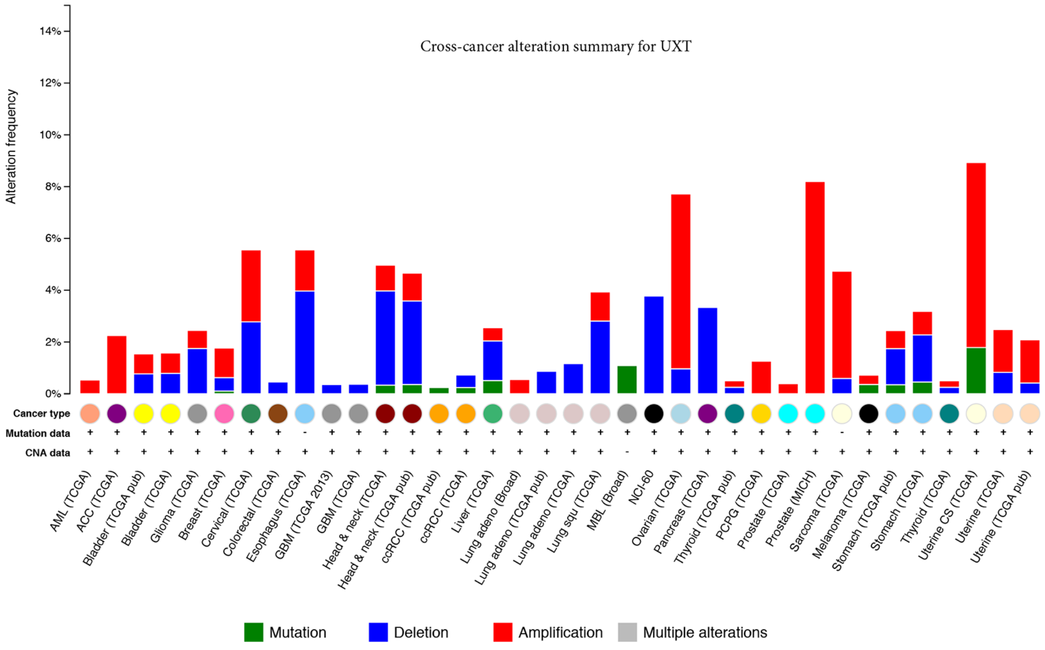

Supplementary Figure S1: Cross-cancer alteration of the *UXT* gene. The information was derived from cBioPortal Cancer Genomics database.

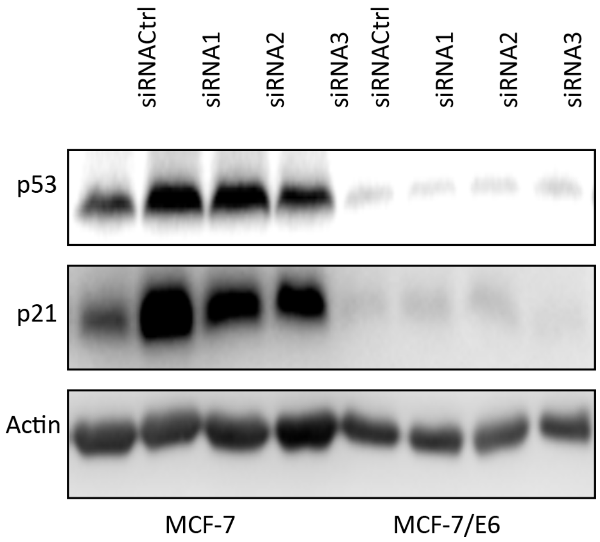

Supplementary Figure S2: UXT downregulation resulted in p53-dependent p21 induction. MCF-7 cells or MCF-7/E6 expressing cells were transfected with either siRNACtrl or 3 independent siRNAUXT sequences. The cells were harvested 48 h after transfection and analyzed by Western with the indicated antibodies.

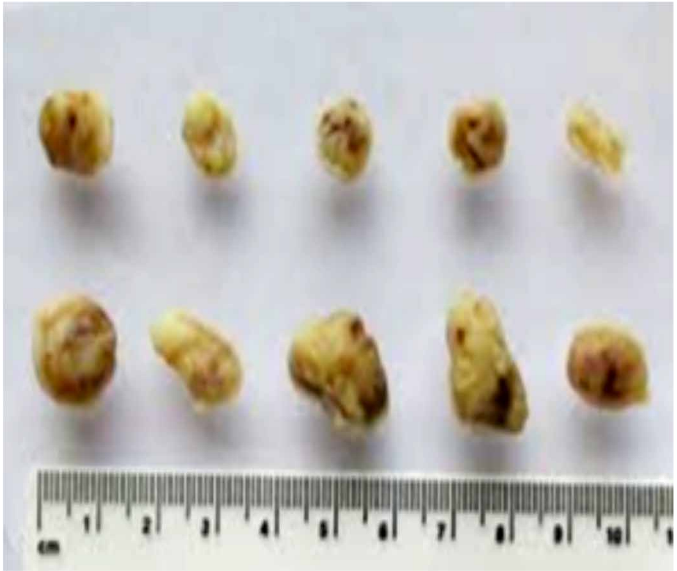

Supplementary Figure S3: Balb C nude mice were subcutaneously implanted with 2 × 10[6] U2OS_EV or U2OS_UXT cells. The tumors were harvested and their images were shown.

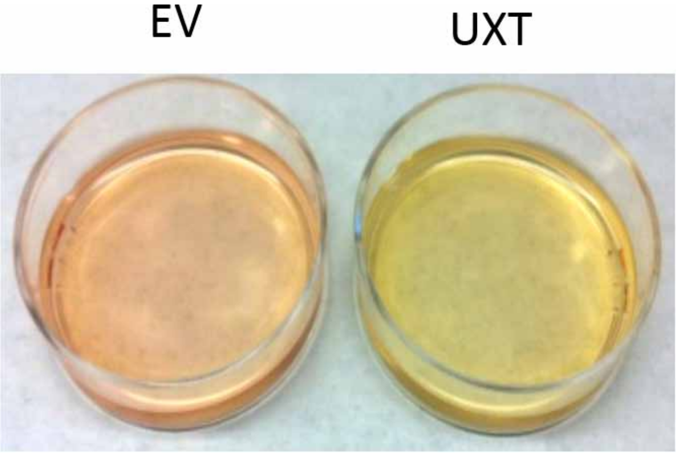

Supplementary Figure S4: U2OS_UXT cells produces more acidic metabolites. The images of culture dishes of U2OS_EV or U2OS_UXT cells were taken 24 h after replating.

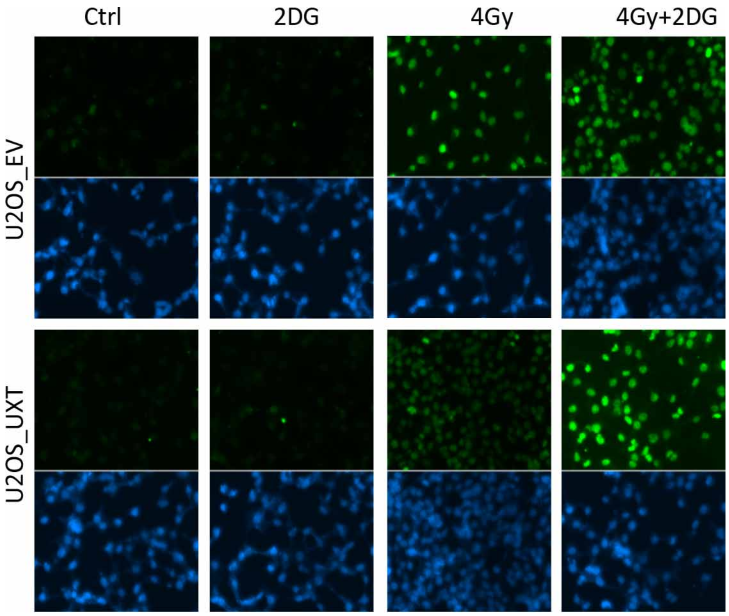

Supplementary Figure S5: A critical role of glycolysis in modulation of radiation sensitivity of U2OS_UXT cells. U2OS_EV or U2OS_UXT cells were pre-treated with vehicle or 2-DG (2 mM) for 6 h followed by irradiation at a dose of 4Gy. The cells were fixed 1 h after irradiation and stained with gH2AX. Representative images were shown.
